# Has the concept of systems consolidation outlived its usefulness? Identification and evaluation of premises underlying systems consolidation

**DOI:** 10.12703/r/11-33

**Published:** 2022-11-22

**Authors:** Morris Moscovitch, Asaf Gilboa

**Affiliations:** 1 Department of Psychology, University of Toronto, Toronto, ON, Canada; 2 Rotman Research Institute, Baycrest, Toronto, ON, Canada; 3 Toronto Rehabilitation Institute, University Health Network, Toronto, ON, Canada

**Keywords:** Memory consolidation, Hippocampus, Cortical plasticity, Schema, Gist, Event Memory

## Abstract

Systems consolidation has mostly been treated as a neural construct defined by the time-dependent change in memory representation from the hippocampus (HPC) to other structures, primarily the neocortex. Here, we identify and evaluate the explicit and implicit premises that underlie traditional or standard models and theories of systems consolidation based on evidence from research on humans and other animals. We use the principle that changes in neural representation over time and experience are accompanied by corresponding changes in psychological representations, and vice versa, to argue that each of the premises underlying traditional or standard models and theories of systems consolidation is found wanting. One solution is to modify or abandon the premises or theories and models. This is reflected in moderated models of systems consolidation that emphasize the early role of the HPC in training neocortical memories until they stabilize. The fault, however, may lie in the very concept of systems consolidation and its defining feature. We propose that the concept be replaced by one of memory systems reorganization, which does not carry the theoretical baggage of systems consolidation and is flexible enough to capture the dynamic nature of memory from inception to very long-term retention and retrieval at a psychological and neural level. The term “memory system reorganization” implies that memory traces are not fixed, even after they are presumably consolidated. Memories can continue to change as a result of experience and interactions among memory systems across the lifetime. As will become clear, hippocampal training of neocortical memories is only one type of such interaction, and not always the most important one, even at inception. We end by suggesting some principles of memory reorganization that can help guide research on dynamic memory processes that capture corresponding changes in memory at the psychological and neural levels.

## Introduction

Recent empirical and theoretical developments have led to a burgeoning interest in memory engram (trace) formation and consolidation^1–5^. However, many of the underlying premises guiding research and theory on memory systems consolidation have either not been stated explicitly or have not been examined critically. Here, we identify and evaluate what we believe are the crucial premises in the hope that they will lead to a more clear-eyed approach to research on memory phenomena that are described within a systems consolidation framework. At the heart of this critical examination is the idea, first enunciated by Burnham^6^, that memory consolidation is as much a psychological process as a neural one and that an appreciation of both is necessary if we are to have a full understanding of the phenomenon^7,8^.

## Historical background and definitions

The term “consolidation” began as a psychological construct to describe an underlying, time-dependent process that enables learning or memory to become stabilized so that it is relatively immune to disruption or loss by subsequent, interfering events^9,10^. Once the sensitive period for consolidation has passed, interfering events lose their efficacy and memory is stable. In the initial, behavioural experiments, the consolidation period was on the order of minutes.

Investigators noted a similar temporal relationship between brain damage and memory loss, in which memory for prior events was lost or impoverished if they occurred close to the onset of trauma, whereas memory for more remote events was relatively unaffected. Linking the evidence from brain damage to that in the psychological laboratory, researchers speculated that the temporal gradient that was observed in both was related to the time it took memories to become consolidated. The discovery that damage to the medial temporal lobes (MTL), and HPC, in particular, led to a temporally graded retrograde amnesia of about 3 to 11 years^11,12^, placed the HPC at the centre of neurobiological theories of memory consolidation^13–16^.

The time course of the temporal gradient caused by brain damage, however, was orders of magnitude larger than that observed in the initial, behavioural experiments and in subsequent experiments in which the disruptive event could be electroconvulsive shock or neurochemical blockade^7,15,16^. To reconcile these different time courses, it was suggested that there might be at least two consolidation processes. One process is rapid and operates at the cellular or synaptic level. Termed *cellular or synaptic consolidation*, it consists of a cascade of cellular and synaptic neurochemical events initiated by learning and ending within hours at most. There is ample neurobiological evidence for cellular synaptic consolidation, which is beyond the scope of the present review. Nonetheless, aspects of the underlying neurochemical events that confer stability are ongoing for the duration of a memory’s life^17^. Moreover, studies on reconsolidation demonstrate that presumably consolidated memories can themselves be disrupted, leading to destabilization of the memory trace^18^. Although we now know that multiple forms of cellular consolidation operate over extended durations, originally an additional process was hypothesized in order to account for longer durations of memory lability that could not be explained by immediate cellular processes. The other, more gradual process was termed *systems consolidation*^19^. It is sustained over much longer periods of time, sometimes decades, and involves the reorganization of memory at the level of large-scale neural systems. According to the traditional view, in systems consolidation, acquisition, retention, and retrieval of declarative memory (episodic and semantic) are initially dependent on the HPC, but with time and experience, memory is consolidated in extra-hippocampal structures, so that memories can be retained and recovered independently of the HPC and related structures of the extended hippocampal system^15,20–22^.

## Standard consolidation theory or model and its derivatives

**Figure 1.**
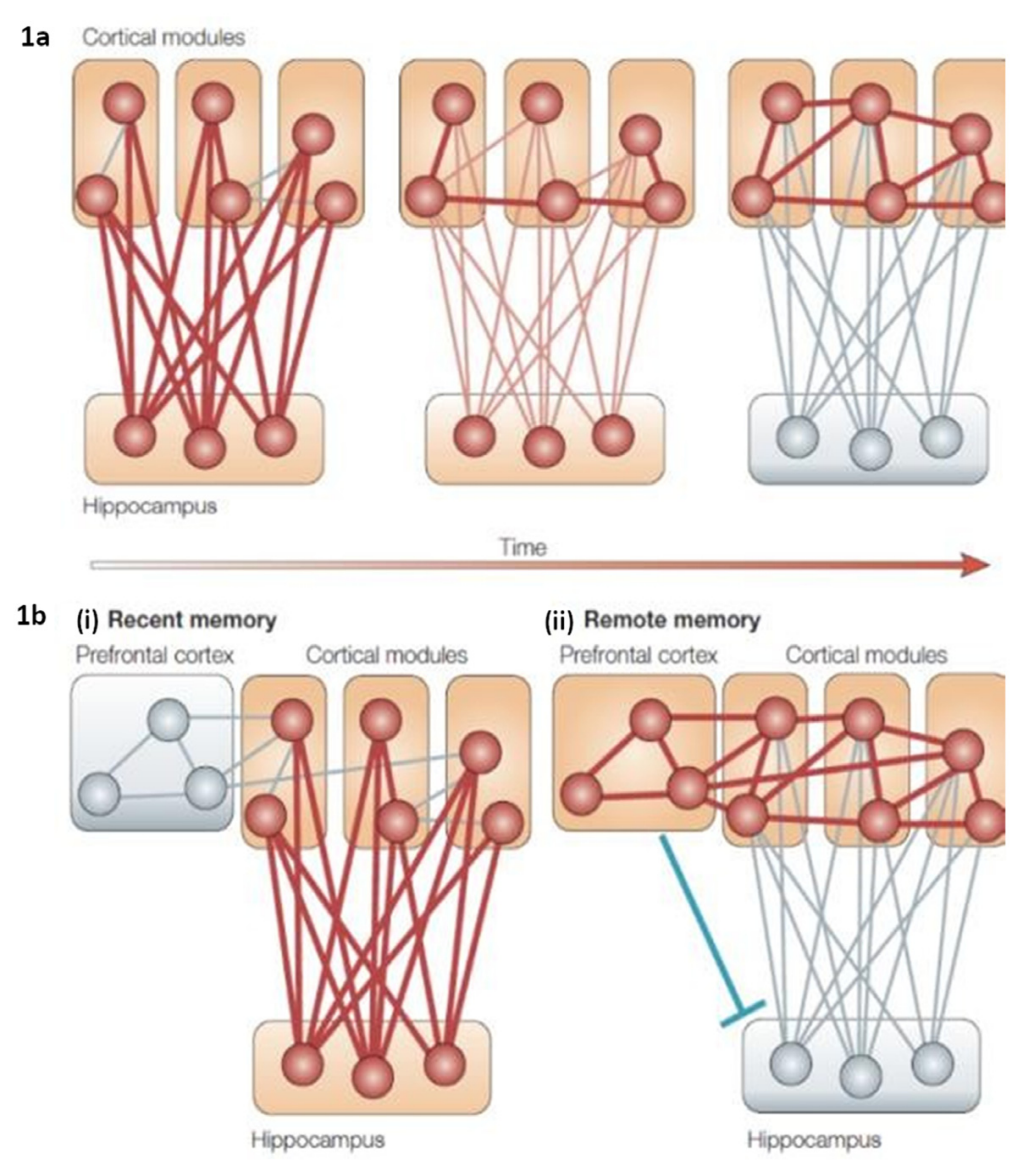
Premises of standard consolidation theory. (**a**) Traditional standard consolidation model. Hippocampal-neocortical connections formed at encoding are weakened with time as neocortical connections are strengthened. (**b**) Modified model in which hippocampal-neocortical connections mediating recent memory are replaced by prefrontal neocortical connections for remote memories, accompanied by frontal inhibition of hippocampal connections. The thicker the line, the stronger the connection. Lines and nodes that are gray have lost their functional contribution. Background premises common to all models and theories of memory and systems consolidation^1–4,8,20–22,28–34^. [Adapted by permission from Springer Nature: Springer Nature, Nature Reviews Neuroscience, The organization of recent and remote memories, Paul W Frankland and Bruno Bontempi (2005) https://doi.org/10.1038/nrn1607^27^]

These observations formed the basis for the standard model of memory consolidation, a version of which is displayed in Figure 1. Neuropsychological theories of declarative memory formation all assume that the HPC binds into a hippocampal-neocortical ensemble (memory trace or engram), the neuronal pattern that underlies the content, context, and experience of an event^23^. The sparsely-coded hippocampal neurons in this ensemble serve as a pointer or index to the distributed neocortical representations of the engram^24,25^. At retrieval, an internally generated or externally driven cue interacts with the hippocampal index, which, in turn, reactivates the ensemble to yield a detailed memory of the event. According to the standard model of systems consolidation, over time, the links among the neocortical elements of the ensemble that constitutes the content of the memory trace, guided/reinforced by the HPC, are strengthened to the point that they can be reactivated without hippocampal input. This marks the end of the systems consolidation process, at which point memories are retrieved directly from the neocortex and independently of the HPC^20,23,26,27^.

## Premises underlying standard consolidation theory and its derivatives

Although consolidation was first a psychological construct, some of the psychological phenomena from which the concept evolved were neglected as neurobiological approaches to studying memory consolidation evolved. As a result, systems consolidation became primarily a neural construct, and theory and experiments typically focused on the neurobiological processes by which memories become independent of the HPC and come to rely on the neocortex. Figure 1 captures the elements of the standard model well and helps us identify the set of premises underlying our ideas of systems consolidation^1–4,8,20–22,28–34^. Some of the premises have been stated explicitly, but others are assumed implicitly yet play a major role. Here, we identify what we consider to be these major premises.

Although each of the premises is stated separately, it will become apparent in the ensuing discussion and evaluation of the premises that many of them are interrelated.

1.     *Binding hub (common to all memory theories)*: The HPC binds co-activated neocortical (and other) neurons that represent the experience of an event into a hippocampal-neocortical ensemble (memory trace or engram).

2.     *Index/pointer (common to all memory theories)*: The HPC acts as an index or pointer, and possibly a spatial scaffold, whose activation reinstates the event in memory.

Premises of the standard model of systems consolidation:

3.     *Initiation*: Memory consolidation begins with the presentation of the to-be-remembered event.

4.     *Gateway*: The HPC is the gateway, or the most efficient pathway, to neocortical representations of all declarative memory.

5.     *Strengthening of neocortical connections*: With time, and replay, the connections among the neocortical elements are strengthened until they are sufficiently strong so that activating any of them can reactivate the entire ensemble without help from the HPC. In the modified model (Figure 1b), connections to the prefrontal cortex are also strengthened. Concurrently, hippocampal binding is diminished. It is these processes that constitute *systems consolidation*.

6.     *Time-limited*: The role of the HPC in reactivating the neocortical component of the memory trace is time-limited. As a result, there is a *temporal gradient* of retrograde amnesia with recently acquired, but not fully consolidated, memories being more vulnerable to disruption than more remote, consolidated memories.

7.     *Equivalence*: All declarative memories, whether event-specific and detail-rich (episodic) or generalized (semantic), undergo the same process of systems consolidation and achieve the same outcome, namely independence from the HPC.

8.     *Comparability*: According to the standard model, the neocortical elements mediating event-specific memories remain unchanged throughout the consolidation process (Figure 1). Except for strengthening of connections among the neocortical elements and loosening those to the HPC, the event-specific memory represented in the neocortex retains its detail-rich characteristics, whether or not it is dependent on the HPC for retention and retrieval. Conversely, generalized memories supported by hippocampal and neocortical representations are also indistinguishable from one another. In some versions of the standard model, the HPC, in addition to helping strengthen neocortical connections, both supports and promotes the reorganization of neocortical connections, although the relation of such reorganization to changes in psychological representation remains unspecified.

9.     *Substitution*: Post-consolidation retrieval can be implemented by activating one of the neocortical elements directly, leading to the reactivation of the entire ensemble. Alternatively, activation of the neocortical elements that represent the content of the memory may be mediated by another neocortical structure (see prefrontal cortex in Figure 1), which plays a role comparable to the HPC, effectively replacing it once consolidation is completed. The primary candidate is the ventromedial prefrontal cortex (vmPFC).

10.     *Unidirectional*: Systems consolidation is unidirectional, proceeding from the HPC to the neocortex.

11.     *Non-reversible*: Systems consolidation and memory dynamism end when stable cortical traces are created. Once consolidated, the process cannot be reversed. Retrieving memories does not make them more susceptible to interference or loss.

Premise 8 (comparability) has been a major point of contention for the past three decades and therefore needs elaboration. In the interest of simplicity, the figure captured one aspect of the standard theory but, in the process, eliminated a more nuanced view of memory as dynamic, even by proponents of the standard model. Perhaps a better way of stating Premise 8 is that memories still dependent on the HPC are fundamentally similar to those that can be retained or retrieved independently of it. They retain the same detail-rich experiential characteristics that hippocampal event-specific memories have and the same informational detail that event-general memories have throughout the consolidation process.

## Evaluation of premises

In the remainder of the article, we evaluate each of the premises in light of recent evidence. Here, we highlight only the studies that we believe are representative of a larger body of evidence or make a particularly salient point. For more extensive, recent reviews, we refer the reader to Gilboa and Moscovitch^8^, Gilboa and Marlatte^35^, Gisquet-Verrier and Riccio^36^, Hebscher *et al*.^37^, Moscovitch and Gilboa^38^, Sekeres *et al*.^7^, and Sutherland *et al*.^39^ for views consistent with our position and to Fernandez and Morris^40^ and Squire *et al*.^41^ for views opposed to it.

We begin with Premises 5 (strengthening neocortical connections) and 6 (time-limited), which are tightly linked and form the core of the standard model or theory of systems consolidation (SCT), namely that by strengthening neocortical connections of the engram, it becomes consolidated over time and independent of the HPC^20,41^. As a result, memories can be retained and recovered without engaging the HPC. Although there was early evidence that hippocampal damage led to temporally extensive and sometimes profound memory loss^20,42–45^, it was not until Nadel and Moscovitch^29^ proposed their multiple trace theory (MTT) that opposition to SCT became more widespread. A crucial element of MTT is the emphasis on the importance of taking into consideration psychological representations, in particular, the distinction between episodic and semantic memory (see Kinsbourne and Wood^46^ as precursors). MTT posited that episodic memory is always mediated by the HPC, no matter how long ago it was acquired. By contrast, semantic memories, which capture statistical regularities among episodes, among other of its aspects, are represented in the neocortex independently of the HPC, sometimes fairly rapidly after acquisition^47^ but more often after a prolonged period of consolidation.

Evidence in favour of MTT began to accrue from studies of patients with various neural or psychiatric disorders associated with hippocampal damage or dysfunction^7,38,48,49^, even if the damage was confined to small components of the HPC, such as its subfields^50,51^ or projections^52^, and to regions of the extended hippocampal system^53–56^ (see reviews by Winocur *et al*.^30,57^ and Sekeres *et al*.^7^). In all cases, remote event-specific memory loss was temporally extensive, often without a gradient, whereas generalized memory was relatively spared (but see Bayley, Hopkins, and Squire^58^; Kirwan, Bayley, Galvin, and Squire^59^; and Squire *et al*.^41^). The same held true for loss of context-specific memories in rodents after lesions of the HPC or its disruption by neurochemical or optogenetic procedures, whereas context-general memories were relatively spared^60–62^ (see reviews in Jasnow *et al*.^63^, Sekeres *et al*.^7^, and Moscovitch and Gilboa^38^).

Converging evidence from neuroimaging studies reinforced these findings in humans and rodents. Functional neuroimaging studies in humans showed that the HPC was activated by the retrieval of event-specific memories no matter how old they were^64–68^. (See Moscovitch *et al*.^48^ and Moscovitch and Gilboa^38^ for review.) Comparably, studies in rodents reported that early gene expression in the HPC was reduced for both recent and remote context-general memories. This evidence is consistent with hippocampal involvement in representing context-specific but not context-general memories regardless of age^69–71^. Early notions of temporal gradients in amnesia distinguished episodic (event-specific) and semantic (generalized) memories, suggesting that only the former always depended on the HPC. When more sensitive measures of semantic representations are used, patients with MTL damage demonstrate deficits in lifelong semantic knowledge, including vocabulary specificity and richness^72,73^, specific knowledge of geography and famous personalities^74^, knowledge of semantic narratives^75^, and aspects of personal semantics^76^. Thus, the nature of representations (rich, detailed, and relational) rather than the age and type of memory (episodic vs. semantic) is what appears to drive hippocampal involvement.

Thus, Premise 6, which stated that hippocampal involvement in memory consolidation is time-limited, is clearly refuted by this evidence (but see Squire *et al*.^41^). Premise 5 may still hold in that neocortical connections may indeed be strengthened by time and experience, but if they are, such strengthening is not sufficient to support the retrieval of detail-rich, event-specific memories without hippocampal involvement.

These findings also refute Premise 7 (equivalence) in that the process of consolidation does not affect all declarative memories equivalently. The fate of event-specific and detail-rich episodic and semantic memories appears to be fundamentally different from that of generalized semantic memories (but see below for a critique of systems consolidation accounts of semantic representations).

Since MTT was proposed, there have been notable advances in our understanding of memory and its neural correlates. In particular, the distinction between details (verbatim) and the gist in event-specific memory has come into prominence^57,77,78^, as has the distinction between categorical knowledge and schemas in semantics^8,35,79,80^. These advances have led us to use the more neutral terms detailed and gist event-specific memory representations (see Rubin and Umanath^81^). *Gist* refers to a summary of what happened at a particular birthday party bereft of most details (e.g., *Sam brought out the birthday cake*). We reserve the term *episodic memory* to refer to detailed, perceptually-based relational representations of high specificity that also includes phenomenological re-experiencing of a particular event (e.g., details of a birthday party: descriptions of the cake, who attended and what they wore, and so on, coupled with a sense of personal time travel). As noted below, the information comprising episodic memory is not limited to detailed, perceptual representations, but they must be included for the memory to be considered episodic. Likewise, with respect to semantic memory, we distinguish between event-general schemas and abstracted ‘encyclopedic’ semantic knowledge, such as categorical knowledge. *Schemas* refer to what is common across similar events (e.g., what happens at a typical, rather than a particular, birthday party). We retain the term *semantic memory* to refer to categorical knowledge or meaning (what ‘birthday party’ means). Another way of conceptualizing these different types of memory is to distinguish between event (context)-specific and generalized memory. Event-specific memories are further characterized by their detail richness, from detail-rich to gist, and generalized memories may be general event schemas or more abstract semantic categories.

At the neural level, semantic processing engages the lateral prefrontal cortex, the medial prefrontal cortex (mPFC), and anterior temporal and inferotemporal cortices but to different degrees; the mPFC is more strongly implicated in schema processes, whereas temporal cortices are more strongly associated with semantic processes^7,82–87^. The HPC, too, is no longer treated as a unitary structure, and evidence suggests that event details are mediated by the posterior regions and event gist by the anterior HPC^8,79,88^. As the DG and CA3 subfields have greater representation in the posterior HPC and CA1, in the anterior, there has been some indication that these subfields are more implicated in processing details (specificity) and gist, respectively^67,89–91^. (For review, see Jasnow *et al.*^63^ and Moscovitch and Gilboa^38^).

We focus on the HPC and mPFC, the main structures mentioned above, as they seem to be hubs of more distributed networks implicated in different types of memory which include the angular gyrus, anterior and inferior temporal lobes, and thalamus, among others. The function of these related structures in the networks is not as clearly delineated with respect to different types of memory as the ones on which we’ve focused. One structure whose role in memory dynamics over time has been more extensively studied is the thalamus. Consistent with its anatomical location, the thalamus plays a central role in memory formation and representation. Patterns of retrograde amnesia in patients with thalamic involvement are very variable^92^. Focal thalamic lesions can lead to ungraded extensive retrograde amnesia for detailed event memory, short-duration retrograde amnesia, or intact memory for pre-injury events. More extensive retrograde amnesia may be related to the disconnection of thalamocortical (mostly thalamofrontal) projections, as seen, for example, in Korsakoff syndrome^92^. Experimental studies show that for detailed, context-specific memories, the thalamus mediates vmPFC-hippocampal interactions at both short^93–95^ and long^96^ delays. For more generalized forms of memory, which tend to be more dominant at long delays, thalamic nuclei form part of vmPFC-thalamic-cortical circuits independent of the HPC^95,97,98^. We note that these long experimental delays would be considered quite short when measuring retrograde amnesia gradients (days to weeks) and that the extent to which cortico-thalamic-cortical circuits are involved in forming new generalized memories at encoding is unknown. For a more in-depth discussion of these related structures, we refer the interested reader to Gilboa and Moscovitch^8^ and Moscovitch and Gilboa^38^.

Anticipating these more nuanced accounts of functional neuroanatomy, Winocur and Moscovitch^57^ and Sekeres *et al*.^7^ extended MTT by advancing their trace transformation theory (TTT) to account for the dynamic nature of memory by proposing that there is a close (lock-step) correspondence between psychological and neural representations of memory with time and experience. According to TTT, the different forms of memory can coexist and interact with one another, and the ones expressed in behaviour are determined not only by time and experience but also by the demands of the task, the goals of the individual, and likely all other factors that traditional studies on memory formation and retention have identified as important. Crucially, for whichever form of memory is expressed, there is a corresponding neural correlate that mediates it. Thus, at the heart of TTT is the *principle of functional-neural isomorphism* which states that “representations that differ from one another must necessarily be mediated by different structures (collections of neurons), and representations mediated by different structures must necessarily differ in some fundamental way from one another”^99^ (p. 109).

Its corollary is that there is neural-psychological representation correspondence (NPRC), namely that each type of representation is mediated by its corresponding structure and vice versa. If detail-rich memory is mediated by the HPC, this relationship should hold regardless of whether the memory occurred recently or long ago, as described above, or even whether it is episodic or detail-rich semantic^72,74,100–102^. Conversely, these principles suggest that if there is a change in the mediating structure, which sometimes occurs as memories age, there should also be a change in the nature of the psychological representation^8^.

The correspondence, however, can take forms other than those linking a psychological representation to a particular structure. For example, the *level* of activation of one or another structure may not track changes in psychological representation, but such correspondences may occur with the *pattern* of activation in that structure or across a distributed network of structures, which can be delineated by multivariate analyses. The interpretation of correspondences between neural and psychological representations in the memory domain is not fundamentally different from the interpretation of these correspondences in other domains, such as perception, problem-solving, or language. The point we wish to emphasize is that the nature of the psychological representation must be considered if we are to understand the nature of the neural representation and vice versa. For example, if two psychological representations are not distinguished from one another at a particular site, such as the HPC, we would conclude that the HPC is not sensitive to those differences. There should, however, be another structure, or network of structures, whose level of activation or pattern of activity can account for those psychological differences.

The evidence, consistent with these principles, undermines Premises 8 (comparability) and Premise 9 (substitution). Representations of event-specific memories in the neocortex are not comparable to those in the HPC. Neocortical memory representations are impoverished in detail, specificity, and vividness but are equivalent or even superior to hippocampal representations in core semantics and schemas, as seen in behavioural and functional magnetic resonance imaging (fMRI) studies in patients with damage or dysfunction of the extended hippocampal system and in fMRI studies of neurotypical people^103,104^. The same holds for rodents whose context-specific memories are compromised in favour of context-general memories following HPC lesions or disruption and in studies measuring hippocampal and neocortical activation^7,63,90^. If event-specific memories retain their detail, vividness, and specificity, the HPC is implicated no matter how long ago the memory was acquired. As a side note, in all the human research described above, semantics and schemas were acquired before the HPC was damaged, leaving open the possibility that the HPC is needed for their acquisition, as traditional models of consolidation predict. We consider this possibility in our discussion of Premise 10 below and note that there are many instances in which schemas and semantics can be acquired relatively normally without the HPC, although in other instances the HPC seems necessary^105–108^. It is not yet clear what accounts for these differences, but there is enough evidence to suggest that how information is encoded and retrieved may be determining factors, as well as the type of schemas and semantics^37,71,74,108–111^. For example, concepts that are quite detailed, and even context-defined, rely on the HPC in a manner similar to episodic memories, while core aspects of semantic memory may not implicate the HPC^72,74,100,105,112^.

In a minority of studies, neocortical event-specific representations are reported to be equivalent in quality to hippocampal ones^113,114^. The source of this discrepancy, however, has yet to be determined, but one possibility is that measures of richness, vividness, and event specificity in those studies have been compromised (see discussion in Sekeres *et al*.^7^ and Moscovitch and Gilboa^38^).

NPRC is also seen along the long axis of the HPC and its subfields. Highly detailed, event-specific memories depend more on the posterior (dorsal in rodents) than anterior (ventral in rodents) HPC, and on DG/CA3 subfields more than on CA1 subfields, whereas the reverse is true for event-specific gist-like representations^67,88,89,115–117^. Damage to the mPFC, implicated in processing schemas, and to ventral HPC and CA1 implicated in processing gist, impairs retention and retrieval of schema and gist representations of recent and remote memories^118–120^. Detail-rich aspects of memory can remain relatively preserved if supported by proper cuing^121,122^. By contrast, when the HPC is damaged or dysfunctional, detail-rich event memory, whether recent or remote, remains impoverished, even though the mPFC is intact^7,8,30^. Such findings are contrary to Premise 9, which posits that the mPFC can serve as a substitute for the HPC in activating detailed, mnemonic representations; it appears that the vmPFC cannot replace hippocampally-supported representations, no matter how remote they are.

The correspondence between psychological representations and their neural correlates is observed both when memories are newly acquired and when they age. Thus, what determines whether or not there is a change in neural representation with time is not the age of the memory, as traditional systems consolidation theories posit, but the nature of the memory representation that is being expressed.

According to TTT and NPRC, the reverse should also hold, namely that changes in the neural correlate being activated should be accompanied by corresponding changes in the psychological representation being expressed. This is seen most dramatically in re-consolidation^123,124^. Re-consolidation refers to the condition in which the synaptic consolidation process is reversed, such that a memory that appears to have become independent of the HPC with time, once again becomes dependent on it after the organism is re-exposed to the training context or relevant aspects of it. Using rats, Winocur *et al*.^125^ showed that the memory that was independent of the HPC was context-general, but once re-exposure occurred, it became context-specific; this pattern is consistent with the return of hippocampal involvement and dominance of expression of context-specific over context-general representations. These findings, along with similar ones^126^, refute Premise 11 that systems consolidation is *not reversible* and, in fact, show that memory consolidation, in general, may be reversible. These findings also, to an extent, refute Premise 10, that systems consolidation is *unidirectional*, always proceeding from the HPC to the neocortex, particularly the mPFC.

Additional evidence against Premise 10 comes from studies showing that the mPFC can contribute to the formation of HPC-dependent memories. Once schemas are acquired, the formation and retention of HPC-dependent memories proceed more rapidly in rodents and humans^37,111,127–131^. Even without evidence of pre-existing schemas, intact mPFC and anterior temporal cortex facilitate the formation of long-lasting hippocampally-dependent episodic memories^132–134^, with the extent of functional connectivity between these regions and the HPC being related to memory formation and retention^135–138^.

To the extent that schemas are reinstated prior to stimulus presentation (a birthday party schema is reinstated prior to all the events that occur at the party), it can be argued that memory formation is initiated even before a stimulus is presented^37^ contrary to Premise 3 (initiation).

Once the stimulus is presented, the schema is instantiated and interacts with the stimulus/event to form a meaningful event-specific memory^38,82,83,139,140^.

Evidence from animal studies on memory allocation also points to the idea that the neural conditions for memory formation are in place before stimulus presentation^141^. The neurons that are most highly activated prior to an event are allocated to form the engram for that event.

Similar results are reported in fMRI studies in humans showing that the greater the representational similarity between pre-encoding rest and encoding in the HPC and related structures, the better the memory for the encoded event^142^.

Studies related to Premise 10 (unidirectionality) also cast some doubt on Premise 4, that the HPC is the *gateway* for the formation and retention of all declarative memory. We know it is crucial for event-specific memory, but what about for semantic and schematic memory or for schematic and semantic components of memory for specific events? Evidence from studies in intact humans and rodents suggests that schemas, mediated by the mPFC, and gist, mediated by anterior HPC and CA1, can be represented early in processing without reliance on those regions of the HPC that code for detail and specificity^37,90,91,130,143,144^. In line with our ideas, evidence from recent behavioural studies^145–149^ shows that schemas are formed at acquisition concurrently with detailed contextual representations, but their influence on memory expression depends on a variety of factors, including task demands and the strength of the different representations over time^150^.

This eclectic view of memory reorganization does not deny the possibility that even if neocortical representations are formed concurrently with hippocampal ones, under some circumstances, the HPC may still be needed to stabilize those representations over time^5,7,151,152^ (but see 153) with sleep playing a crucial role^154^ (but see 32 for a different interpretation of the benefits of sleep) or that the HPC may be needed to reinstate them at retrieval^7,155^.

Several studies of language acquisition suggest that aspects of vocabulary that are consistent with prior linguistic knowledge can be rapidly cortically acquired with little hippocampal engagement^156–158^. Likewise, patients with hippocampal damage and poor episodic memory can acquire semantic knowledge through a procedure termed fast mapping, in which single or few presentations suffice for memory formation and retention^47^ (but see Smith *et al*.^159^). It is not yet known, however, whether and to what extent the HPC is needed to retain such memories over long intervals, as semantic memory acquired through fast mapping may be highly susceptible to interference^84^. A partial answer is provided from studies of patients with developmental (episodic) amnesia caused by neonatal MTL damage. Such patients have relatively preserved semantic and schematic knowledge, which is often sufficient for them to complete at least high school, suggesting that even long-term retention of schemas and semantics does not rely on the HPC^160^. Interestingly, individuals with developmental amnesia do not show a memory advantage for fast mapping at short delay but do show delayed gains, contrary to controls who show forgetting, helping them catch up despite their hippocampal damage^161,162^. If, however, the HPC is intact at acquisition, the context-specific hippocampal representation may form part of the ensuing neural/psychological representation of schemas and semantics, or it may serve as the link through which schemas and semantics related to the event are recovered. Subsequent damage to the HPC may lead to an impoverished representation of both. Even if the HPC remains intact, the memory for specific details may decline with time and experience, and the semantic/schematic components may become ascendant, leading to the recovery of those latter aspects of the memory independently of the HPC. 

Insight into the underlying processes and mechanisms may be gained from recent developmental studies on the interaction between the ability to form rapid generalizations (e.g., schemas and semantics) and memory for specific events^163^. Whereas in adults, generalizations are tightly coupled with memory for specific instances, children generalize independently event-specific memory^163^. Different processes may be expressed in childhood and adulthood, determined, in part, by the neurodevelopmental trajectories of the structures that mediate these behaviours and their network dynamics^164^. The extent to which these processes could be expressed in adults but are masked by the more dominant HPC-mediated memory processes is unknown.

## Is systems consolidation a viable concept?

These premises, or a subset of them, underlie not only SCT and theories derived from it but also a number of other theories and models of systems consolidation, including some of the most prominent ones in the field, such as complementary learning systems^31,165^ and some *s*chema theories^128,130,132,166^. If, as we’ve argued, each of these premises is found wanting in light of the evidence, then the theories that embrace them should be modified or discarded. But if all the premises underlying many of our theories of systems consolidation are faulty, maybe the fault lies with the concept of systems consolidation itself. A number of theories and models have been proposed that do not rely on these premises, among them distributed reinstatement theory and memory manifold theory^167^, scene and event construction theory^168^, contextual binding theory^33^, and competitive trace theory^34^*.* Their proponents renounce the notion of systems consolidation either explicitly (Sutherland, Lehmann, and their colleagues^167^) or implicitly, as is the case for the others while retaining the notion of synaptic or cellular consolidation (see Moscovitch and Gilboa^38^ for an evaluation of each of these theories). Instead of focusing on the HPC relinquishing its role in memory to the neocortex in retention and retrieval of remote memories, as the defining event of systems consolidation, these theories concentrate on basic processes and mechanisms of memory formation, retention, retrieval, and loss at a neural and psychological level. They then use knowledge of these basic processes to account for changes in memory, and its neural basis, over time and experience. Among the processes underlying such changes are reminding and forgetting, decay and interference, reconstruction and distortion, assimilation and accommodation, which can be studied without being fettered by adherence to a poorly supported notion of systems consolidation that has little to say about most of them. Most importantly, such a framework allows us to examine the various ways that memories can change over time during wakefulness and sleep at a psychological and neural level. As a result, these non-consolidation theories, and the research they inspire, enable a better appreciation and understanding of the dynamic nature of memory, reflected in its changing representations at psychological and neural levels.

## Memory systems reorganization: some principles

Our own view at the moment is to replace the term systems consolidation with the term *systems reorganization* and offer the following principles to guide research^8,38^.

1.     *From inception, declarative memories of an event capture the conscious experience of the event, which includes information about its perceptual aspects (details) and its meaning (schemas and semantics). These psychological representations have their corresponding distributed neural representations, termed engrams or memory traces that are bound ensembles of neurons that are active during the experience, and different brain regions code the different aspects of the event. For event-specific detailed and gist memory, the HPC binds all the activated neurons into a sparsely encoded hippocampal representation that serves as an index or pointer to cortical locations where these features and content of the memory are represented. For semantic and schematic representations, new generalized information from the event can be integrated into existing cortical knowledge structures, potentially adding new nodes and modifying existing network connections and relationships^8,35,37^. Time and experience may affect aspects of this memory representation differently.*

2.     *The HPC retains its function in representing event-specific memories, from details to gist, and does not relinquish it to other structures over time. Whether the memory is recent or remote does not matter.* Changes along the long axis of the HPC, from posterior to anterior, and among its subfields, from DG/CA3 to CA1, will reflect, respectively, the prominence of details and gist in memory at different times^7,79^. Event-specific HPC-dependent representations tend to dominate memory expression early on, although event-induced memory integration and modifications to semantic and schematic networks also occur at the outset^8,35,37^. *As event-specific details and gist are lost, the generalized representations that are independent of the HPC and reliant more on the neocortex become more prominent*^8^*.* Post-encoding rest^136,169^ and sleep seem to play crucial roles in these processes, either by protecting memories from interference^32^ or by promoting memory replay, which can serve to either maintain or strengthen episodic memories^154^, integrate them with one another^170^, or hasten the assimilation of new details into schemas^154,171^.

3.     *Memory reorganization is not a unidirectional, time-dependent process. Memories are always in flux, from acquisition onwards.* From inception, there are potentially multiple interactive forms of event representations^7,57^, and the one which is preferentially expressed at any moment is determined not only by the passage of time but by various factors, from forgetting to task demands to motivation, at encoding and retrieval^145,150,172,173^. Studies on the development of memory from infancy to adulthood^164,174–177^ and its progression to old age provide insights into the dynamic, multidirectional processes and mechanisms that mediate memory representations.

4.     *Each of these psychological forms of representations is supported by distinct neurobiological substrates and processes, as posited by NPRC*^8^*, and their interactions during wakefulness and sleep drive memory dynamics for the memory’s lifetime*^7,57,79^. Memory reorganization involves more than the relinquishing of hippocampal involvement to the neocortex over time, which may never occur for some memories (see principle 1 of the memory reorganization perspective). It is a dynamic process of hippocampal-neocortical interactions that determine the organization and expression of memory that begins even before acquisition and potentially continues for a lifetime.
